# Implementation of SEEK in a Children’s Advocacy Center: A Process Improvement Initiative

**DOI:** 10.1097/pq9.0000000000000573

**Published:** 2022-06-23

**Authors:** Megan M. Letson, Farah W. Brink, Alicia Daniels, Sandra Thompson, Kathryn G. Wolf, Nichole L. Michaels

**Affiliations:** From the * The Center for Family Safety & Healing Nationwide Children’s Hospital, Columbus, Ohio; † Department of Pediatrics, The Ohio State University College of Medicine, Columbus, Ohio; ‡ Center for Injury Research and Policy at the Abigail Wexner Research Institute at Nationwide Children’s Hospital, Columbus, Ohio.

## Abstract

Risk factors for child maltreatment are well-described, but clinicians may overlook these risk factors. The Safe Environment for Every Kid (SEEK) model is an evidence-based approach to identifying psychosocial risk factors for child maltreatment. This article describes a quality improvement initiative to implement the SEEK model in a unique pediatric setting, a Children’s Advocacy Center.

**Methods:**

The objectives were to (1) describe the identification of psychosocial risk factors for child maltreatment by implementing the SEEK screening tool with each new family, (2) achieve and sustain a SEEK completion rate of greater than 85%, and (3) achieve and sustain a SEEK follow-up compliance rate of greater than 75%. Structured quality improvement methods, including several plan-do-study-act cycles, were used to implement interventions.

**Results:**

The percentage of caregivers who completed the SEEK questionnaire increased from a baseline of 76% to 86%, which was sustained for more than 2 years, resulting in a better understanding and support of families’ needs. Caregivers completed 3,606 SEEK Parent Questionnaire-R. Mental health concerns and food insecurity were among the most commonly endorsed items. Follow-up compliance increased from 47% to 90%, a level that has been maintained.

**Conclusions:**

While Children’s Advocacy Centers evaluate children with suspected abuse, identifying current stressors in the home and linking families with resources to address their immediate psychosocial concerns can improve short- and long-term outcomes. This initiative demonstrates the feasibility of incorporating consistent screening for psychosocial risk factors for child maltreatment in this busy environment.

## INTRODUCTION

### Problem Description

Child maltreatment is a significant public health problem. Abused and neglected children are at greater risk of injuries, psychological disorders, poor health, and other negative personal and social outcomes.^1–7^ In 2019, an estimated 656,000 children in the United States had at least one substantiated referral to children services for child maltreatment.^8^ This figure is undoubtedly an underestimate.^9,10^ Child maltreatment risk factors are well-described and include parental depression, substance abuse, harsh punishment, major stress, and intimate partner violence in the home.^11^ Given the child welfare system’s narrow focus and limited resources, there is a growing need to identify and address child maltreatment risk factors using other mechanisms.

### Screening At-risk Families

The Safe Environment for Every Kid (SEEK) model was developed with the perspective that pediatricians are uniquely positioned to help prevent child maltreatment through their routine interactions with families.^12,13^ Further, the SEEK model supports the concept that identifying social determinants of health, which are also risk factors for child maltreatment, in a pediatric healthcare setting provides a valuable opportunity to assist at-risk families in a supportive environment. Components of the model include healthcare professional training, a brief caregiver survey to identify psychosocial risk factors for child maltreatment, SEEK algorithms to prioritize and address targeted problems, referrals to behavioral health professionals, and written parent resources.^13–15^ Designed to support and strengthen families of children 0–5 years old, the SEEK program is an evidence-based, cost-effective approach associated with reductions in child maltreatment.^12,14-17^ Over the past decade, the SEEK model has been successfully implemented in various settings, including rural and urban primary care practices and child-care centers.^13,18^

This quality improvement (QI) initiative expands upon existing SEEK research to implement the model in a unique pediatric setting—a Children’s Advocacy Center (CAC). CACs provide specialized services by evaluating children for suspected child maltreatment and serving families at risk for future abuse.^19^ While CACs evaluate children with suspected abuse, interventions administered in the CAC can aid in healing and prevent recurrent maltreatment. In addition, CACs can improve families’ short- and long-term outcomes by identifying current stressors and linking caregivers with resources. National Children’s Alliance, the national accreditation organization for CACs, has a mental health standard that CACs must provide “supportive services for caregivers” with “attention to mental health, substance abuse, domestic violence, and other trauma histories.”^20^ Our CAC did not have a consistent, systematic process to screen families for such concerns, and the SEEK model offered an approach to address this challenge.

This article describes a QI initiative implemented in the CAC at a large midwestern children’s hospital. The CAC employed a collaborative approach utilizing specially trained mental health advocates (MHAs) with advanced social work or counseling degrees in SEEK model administration. The objectives of this QI project were:

(1) To describe the identification of psychosocial risk factors for child maltreatment by implementing the SEEK screening tool with each new family evaluated at the CAC;
(2) To achieve and sustain a SEEK questionnaire completion rate of greater than 85%;
(3) To achieve and sustain a SEEK follow-up compliance rate of greater than 75%.

This study describes the use of the SEEK model in a novel clinic setting and expands its use to include caregivers of school-aged children and adolescents. The SEEK model creators have endorsed but not previously studied this population.

## METHODS

### Description of Study Site and Population

Annually, the CAC evaluates > 1,300 patients referred for evaluation of suspected acute and nonacute child sexual abuse and/or physical abuse by several sources, including children services, law enforcement, emergency departments, primary care providers, and caregivers.

The multidisciplinary team (MDT) CAC model is considered the best practice in child maltreatment investigations. At this CAC, the MDT includes a forensic interviewer, MHA, and medical provider. Patients undergo forensic interviews and medical examinations, while nonoffending caregivers receive education and support services from MHAs. The forensic interviewer performs nonleading interviews of children regarding child maltreatment for medical diagnosis and treatment. The MHA gathers psychosocial information from the family and provides mental health recommendations, making MHAs instrumental in SEEK implementation.

The CAC launched the SEEK program as a QI initiative on July 15, 2017. As this QI project was not human subject research, it was exempt from review by the Institutional Review Board at the authors’ institution.

### Measures

The CAC implemented the SEEK model in this high-risk population to improve the identification of modifiable psychosocial risk factors for future child maltreatment. The SEEK model includes 5 key components: (1) healthcare practitioner training on the SEEK model; (2) SEEK parent questionnaire; (3) use of the reflect, empathize, assess, and plan approach to discuss SEEK questionnaire findings; (4) inclusion of mental health professionals, when available, to aid in assessment and referrals; and (5) distribution of parent handouts.^14^

### Description of SEEK Questionnaire

The validated SEEK Parent Questionnaire-R (SEEK PQ-R) is the centerpiece of the model, allowing healthcare practitioners to screen efficiently for self-reported psychosocial risk factors for child maltreatment. Prior studies have demonstrated that SEEK implementation is a cost-effective way of reducing child maltreatment rates across multiple outcome measures, including fewer reports to children services, fewer instances of possible medical neglect, and less harsh punishment reported by parents.^12,17^ Pediatric health professionals who received SEEK training demonstrated significant improvements in addressing psychosocial risk factors for child maltreatment, including depression, substance use, intimate partner violence, and major parental stress compared with a control group.^21,22^

This QI project used the 2017 revised SEEK PQ-R, consisting of 16 questions to which caregivers can respond “yes” or “no.”^13^ The SEEK PQ-R introduction explains the overall goal of the assessment is to help families and keep children safe. The questionnaire asks nonthreatening questions first (eg, whether the family needs a smoke alarm) before asking more sensitive questions.^13^ SEEK PQ-R screens for food insecurity, harsh punishment, major parental stress, parental depression, intimate partner violence, and parental substance use. Caregivers may also write in other items for which they would like help.

### Implementation

All caregivers of patients presenting to the CAC for initial evaluation of suspected child maltreatment received the SEEK PQ-R to complete. The SEEK PQ-R is voluntary and designed for caregivers to complete in 2–3 minutes.^13^ If multiple children in a family presented, only 1 form was completed by the caregiver. The MHA met with the caregiver to discuss any endorsed responses and provide resources as appropriate. The MHA was also responsible for documenting SEEK PQ-R responses and any resources provided in each patient’s electronic health record (EHR).

### Interventions

Early in implementation, we noted inconsistency in SEEK PQ-R completion; therefore, we assembled a multidisciplinary CAC QI team, including medical providers, a forensic interviewer, an MHA, intake/registration staff, and a QI data analyst. This group met regularly to identify steps for improving consistent administration. The aim was to increase the percentage of SEEK PQ-Rs completed and entered into the EHR for children evaluated at the CAC from 76% to > 85% by December 31, 2017 and sustain this increase for at least 12 months.

**Fig. 1.**
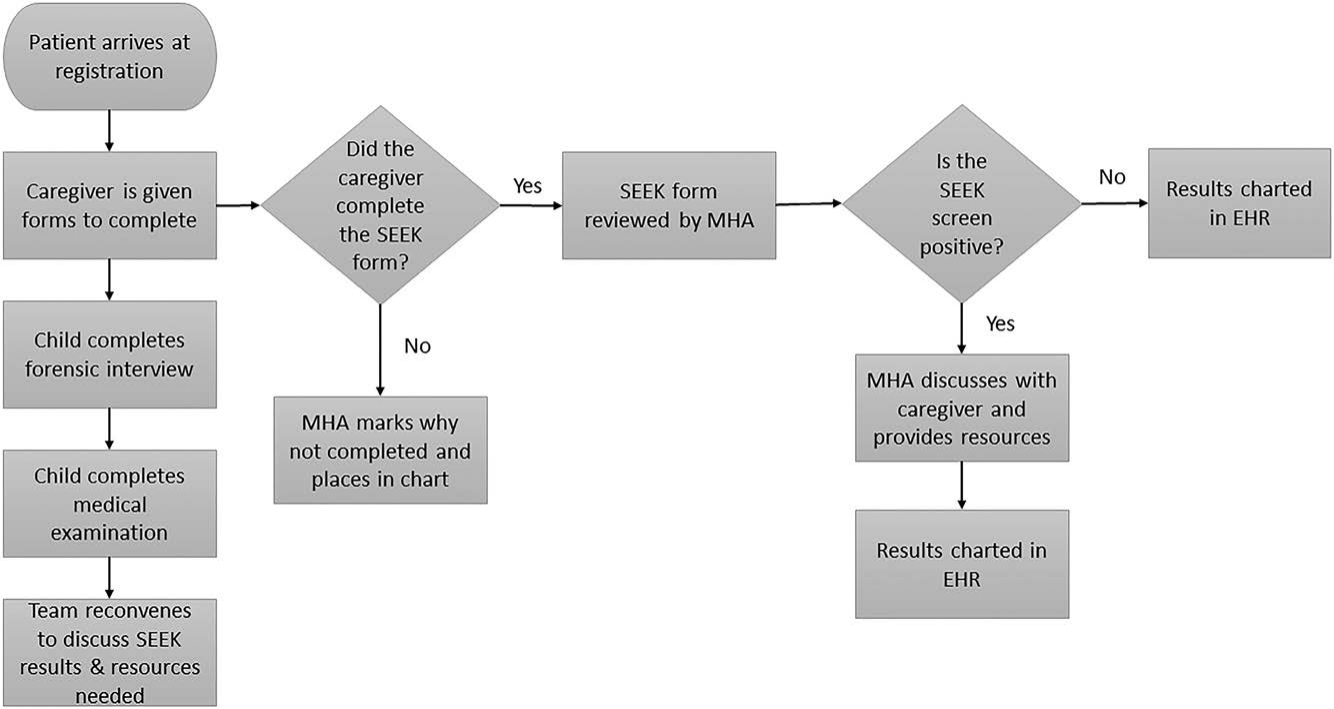
Process map developed by QI team to determine key drivers and identify interventions.

**Fig. 2.**
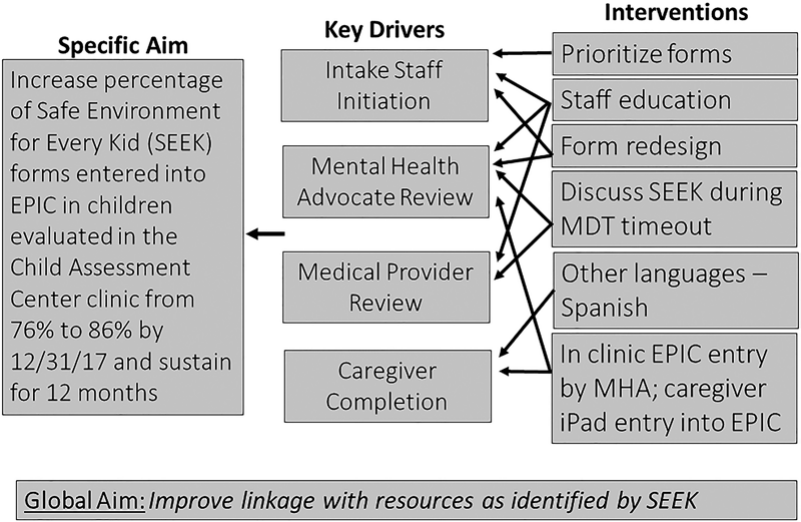
Aim and key driver diagram for initiating the QI project to increase the rate of completion of SEEK questionnaires at the CAC EPIC is the electronic health record used at our institution.

At project onset, the team developed a process map to complete SEEK PQ-Rs (Fig. 1), highlighting necessary steps for questionnaire completion and EHR entry. The process map also helped identify key drivers of different groups within the CAC who were instrumental in SEEK PQ-R completion and/or EHR entry (Fig. 2).

The QI team identified multiple interventions to establish standardized processes for SEEK PQ-R administration and EHR entry. The team prioritized interventions using an Effort/Impact Analysis. Since MHAs were the one clinician type who followed patients through the entire visit, they were influential in creating a standardized workflow and ensuring SEEK PQ-R completion and EHR entry.

The first Plan-Do-Study-Act (PDSA) cycle involved prioritizing paperwork for caregivers. Registration staff gave caregivers multiple forms to complete but did not provide specific guidance about the order of completion, highlighting an opportunity for improvement. By providing the SEEK PQ-R at registration and prioritizing its completion, caregivers had ample time to complete it in the waiting area or private mental health room while waiting to start the assessment. This intervention allowed MHAs to review responses and provide resources during assessments.

During the second PDSA cycle, staff participated in additional education focusing on project goals, benefits, and team roles and responsibilities. Staff received regular feedback about completion rates, facilitating timely recognition of declining completion rates and exploration of causes. Competing duties impacted MHA completion rates and EHR entries; therefore, operation staff began tracking SEEK PQ-Rs and reminding staff to turn in the completed questionnaire or indicate why the questionnaire was incomplete.

**Fig. 3.**
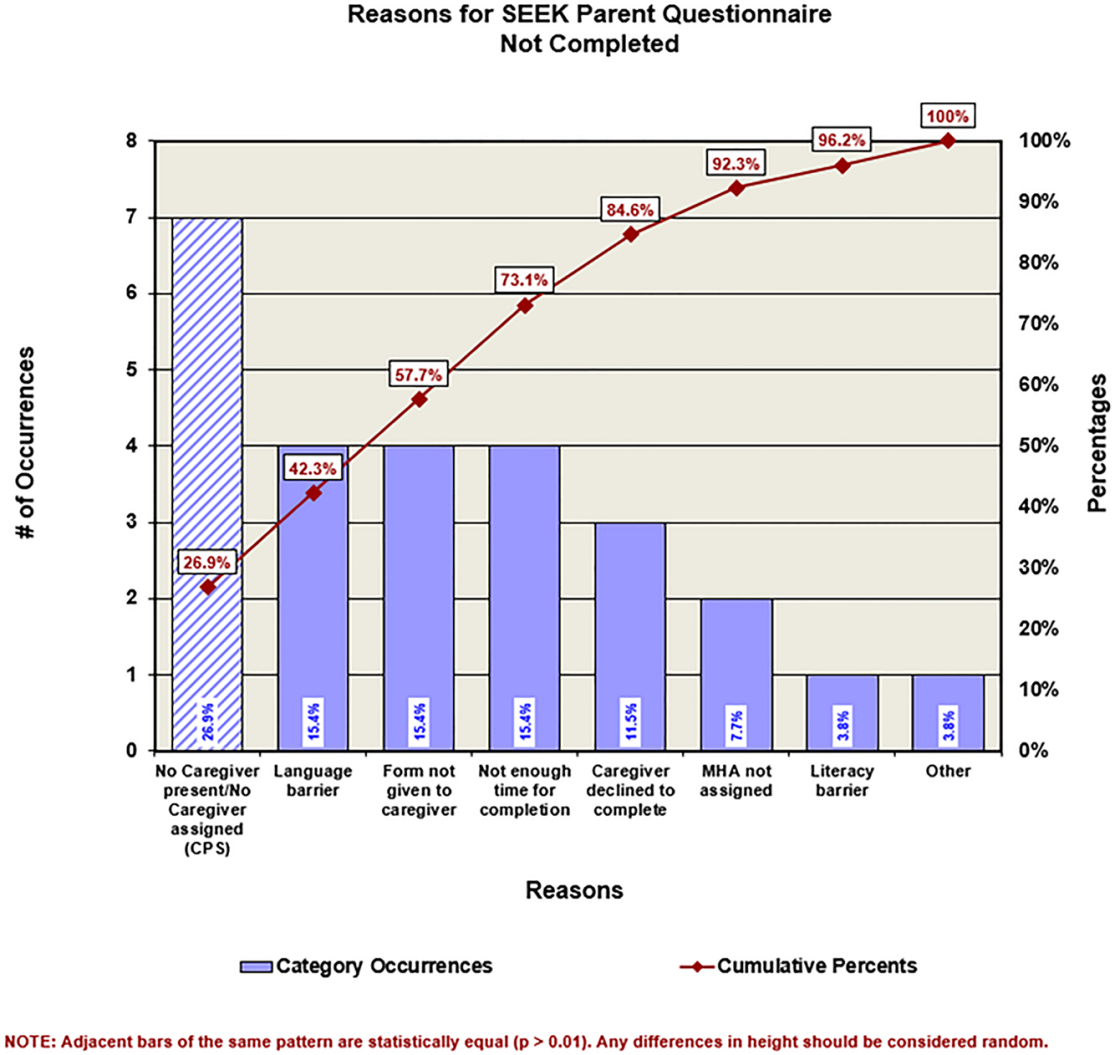
Example Pareto chart showing the reasons that a SEEK form was not completed from July 1, 2017, to November 22, 2017. CPS, Child Protective Services.

Despite interventions focused on process and education, SEEK PQ-Rs were still not consistently being completed and entered into the EHR. Therefore, the third PDSA involved redesigning the questionnaire format. The redesign used colored paper to differentiate it from other paperwork and included a checkbox to note the completed EHR entry. An additional note section allowed MHAs to indicate reasons for incomplete questionnaires. The redesign did not alter SEEK PQ-R questions. Pareto charts summarized the reasons for incomplete questionnaires and facilitated understanding (Fig. 3 for a point-in-time example). The most common reason for incomplete SEEK PQ-Rs was no caregiver was present for the assessment. To ensure safety in acute child maltreatment situations, others, including children services, law enforcement, or another caregiver who is not the residential parent, may accompany a patient to the CAC. Pareto charts helped inform other interventions. For example, language barriers accounted for approximately 15% of the incomplete questionnaires. Initially, the SEEK PQ-R was only offered in English; however, a Spanish version was developed midway through the project. Pareto chart data regarding incomplete questionnaires confirmed that > 85% was an appropriate goal, as barriers to reaching a higher completion rate were out of staff’s control (eg, caregiver not present).

To monitor progress toward increasing completed questionnaires, we compared the number of SEEK PQ-Rs entered into the EHR to the number of caregivers who should have completed the questionnaire. Automated reports provided weekly completion rates; however, the control chart reflected monthly numbers recorded on a p-chart.

#### Follow-up

During assessments, MHAs discussed positive SEEK PQ-R responses with caregivers and provided resources. As a result, these caregivers received follow-up phone calls within 45 days of their visit to discuss linkage with services, additional needs, and barriers. Caregivers qualified for a follow-up call if they answered “yes” to any SEEK PQ-R questions, excluding the first three (needing the Poison Control number or a smoke alarm and identifying smoking at home). The team excluded the first 3 questions because they were not related to psychosocial risk factors for child maltreatment, and the MHAs had already provided resources during the assessment. The SEEK follow-up compliance rate monitored progress toward improving phone follow-up. Follow-up compliance was defined as a completed call with the caregiver or 2 documented attempts to contact the caregiver within 45 days of the CAC visit. Automated reports provided monthly compliance rates, which were recorded on a p-chart.

For all p-charts, baseline data for an initial 6 months were tracked, and a centerline (average) and control limits (± 3 SD, representing inherent process variation) were calculated. Monthly data were entered into the p-chart while holding the baseline constant. We applied statistical process control rules to detect special cause variation versus common cause variation and determine when a centerline shift occurred. *P* values were calculated using Fisher’s exact test and were determined to be significant at *P* = 0.05.

## RESULTS

**Fig. 4.**
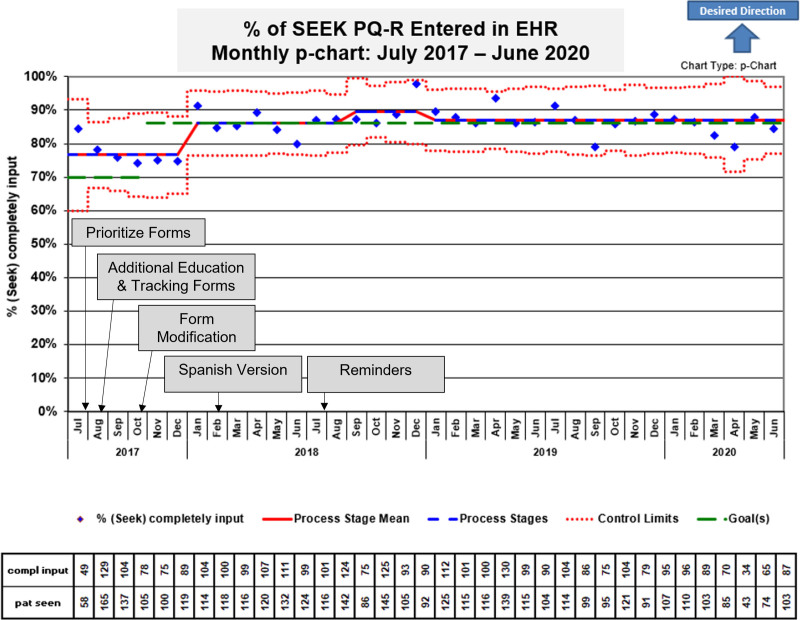
Annotated control chart (p-chart) with the percentage of SEEK questionnaires documented in the EHR from July 1, 2017, to June 30, 2020. A centerline shift was achieved from 76% to 86%. EHR, electronic health record.

Figure 4 is the p-chart demonstrating the percentage of caregivers who completed the SEEK PQ-R and highlights an increase from a baseline of 76% to 86% in the 6 months following initial staff training (*P* < 0.001). This higher completion rate was sustained through mid-2020, with several months reaching completion rates of ≥ 90%. On average, 93 caregivers completed the SEEK PQ-R monthly.

The improved completion rate in January 2018 from 76% to 86% highlights the interventions’ impact. A change in workflow to engage MHAs in ensuring SEEK PQ-R completion and EHR entry was vital to this improvement. Automated reports highlighting missing data helped to sustain improvement. The QI team regularly shared these reports with the team, which informed subsequent interventions. In addition, CAC staff developed data entry reminders, allowing for their active involvement and promotion of a team approach.

Table 1 summarizes SEEK PQ-R responses. Caregivers completed 3,606 SEEK PQ-Rs with 509 (14%) blank responses for each questionnaire item. Mental health concerns and food insecurity were among the most endorsed items, with 23% (724/3,102) endorsing depressive symptoms and 10% (322/3,099) endorsing food insecurity.

**Table 1.** Summary of SEEK Parent Questionnaire-R Responses

SEEK PQ-R Question	Yes (%)	No (%)
Food insecurity	322 (10%)	2,777 (90%)
Harsh punishment	108 (3%)	2,992 (97%)
Extreme stress	766 (25%)	2,335 (75%)
Depressive symptoms	724 (23%)	2,378 (77%)
Intimate partner violence	106 (3%)	2,986 (97%)
Parental alcohol use (> 4 drinks in a day)	95 (3%)	3,012 (97%)
Substance use	56 (2%)	3,049 (98%)

**Fig. 5.**
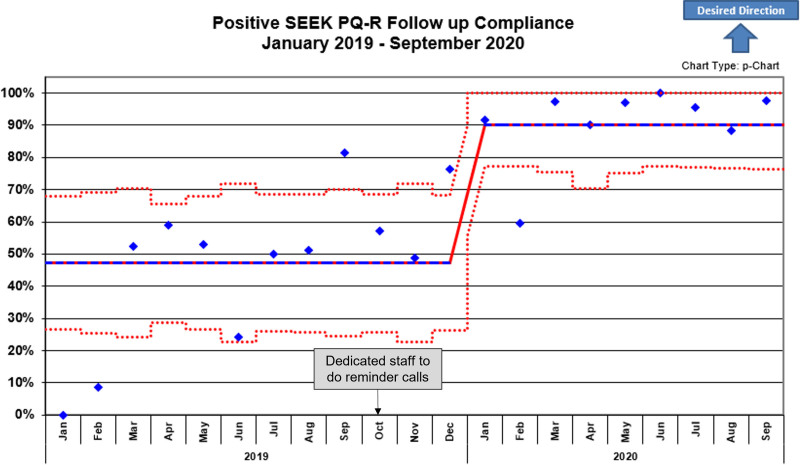
Annotated control chart (p-chart) with the percentage of follow-up compliance (2 attempts or completed within 45 days of visit) from January 1, 2018, to September 30, 2020.

Figure 5 displays the percentage of SEEK-positive caregivers who received followed up, demonstrating an increase from 47% in January 2019 to 90% by January 2020 (*P* < 0.001) with sustained improvement through mid-2020. Several months reached compliance rates of ≥ 95%. On average, 28 caregivers with SEEK PQ-R positive responses received follow-up each month. The increase in the compliance rate highlights the interventions’ impact. Assigning dedicated staff to complete follow-up calls alleviated MHA workload and a clearly defined 45-day completion period aided in achieving and sustaining follow-up compliance.

## DISCUSSION

CACs are uniquely positioned to identify and address psychosocial factors associated with child maltreatment. However, our CAC was not consistently screening for these targeted psychosocial concerns before SEEK implementation. Therefore, the goal of this QI initiative was to create a consistent process change for the administration of the SEEK PQ-R to assess these risk factors and provide resources when applicable.

The SEEK PQ-R was initially designed for a primary care setting, allowing for continuity with families and the ability to reassess ongoing needs periodically. Before implementation, the QI team consulted with the SEEK creators to inform implementation in a CAC setting, given the time-limited contact with families. This project demonstrated it was feasible to implement and sustain a process that administered SEEK PQ-Rs at greater than 85% compliance for more than 2 years in a high-volume CAC setting.

One key factor in successful implementation was the demonstrated benefit to families. Before the consistent screening, the MHAs identified some needs during their sessions; however, the MHA role was largely supportive. SEEK model implementation enabled the identification of social and mental health needs in a structured, efficient manner, allowing MHAs to provide resources to families consistently. Although CAC involvement is time-limited, our MHA model supports short-term follow-up to facilitate linkage with services.

During implementation, establishing process accountability was essential. Several interventions were necessary to improve this process, including regular completion rate feedback, periodic checks on process implementation, and staff education. A flowchart was created in the EHR to facilitate efficient documentation while allowing easy data extraction. Additionally, active participation by MDT members was integral to project success, including the identification of champion leads who provided direct patient care in the CAC. These champion leads identified interventions, provided periodic checks on process implementation, established reliable methods to maintain change, and provided bidirectional feedback and communication between their teams and the QI team. These champions also helped provide stability during periods of staff turnover.

### Challenges and Limitations

SEEK model implementation included challenges. The QI team recognizes the limitation that the SEEK model was developed for a primary care setting. However, we believe that there was a benefit to creating an efficient, standardized approach to identifying and addressing psychosocial concerns in families. Staff did not always recognize the importance of the project, and gaining support was difficult during the initial implementation phases. There were perceptions that the SEEK PQ-R would distract from acute child maltreatment concerns, limiting the MHA’s ability to support families and increasing staff workload if MHAs identified other psychosocial needs. To reframe concerns, the importance of helping families by addressing other critical issues, such as food insecurity or unstable housing, was emphasized regularly. To ease workloads, we developed a resource binder to aid staff in providing information on local housing programs, food pantries, and parenting classes. Despite providing resources, families not linked with services continue to be a concern. We plan to explore barriers further and implement strategies to promote linkage. This challenge highlights the importance of good referral processes and customized handouts.

Another concern was the impact on clinic flow. During the beginning stages, staff perceived that adding the questionnaire to the other required forms inhibited the caregiver’s ability to complete the lengthier paperwork, potentially delaying the MHA’s contact with the caregiver. The QI team reviewed the brevity of the questionnaire with staff; however, initially, it was sometimes not completed due to a reported lack of time. This issue was mitigated by placing the SEEK questionnaire at the top of the registration paperwork.

Ultimately, staff serving as project champions led to a more positive attitude regarding the project’s intent of helping to strengthen families. One champion took ownership of collecting every patient form and ensuring that MHAs identified reasons for incomplete forms. This change bolstered the expectation that caregivers should complete questionnaires unless extenuating circumstances existed. Additionally, an MHA champion embraced the project and modeled utilizing the questionnaire during assessments positively. When staff turnover occurred during the project, new MHAs seemed to accept the intention of the questionnaire and expectation to address psychosocial issues more easily as their onboarding introduced the SEEK program.

### Future Goals

The success of this QI effort has allowed for ongoing work to evaluate mediation of the risks for child maltreatment in this population. Our next goal is to evaluate families’ abilities to link with services and address barriers to service linkage.

## CONCLUSIONS

This initiative describes a systematic QI approach to implementing the SEEK model to assess psychosocial risk factors for child maltreatment. In addition, we demonstrated the feasibility of incorporating this screening process in a busy CAC setting. This finding may be beneficial to other CACs and facilitate compliance with CAC accreditation standards for mental health services to support the safety of children and reduce the risk of future abuse.

## DISCLOSURE

The authors have no financial interest to declare in relation to the content of this article.

## ACKNOWLEDGMENTS

Assistance with the study: The authors would like to thank the Nationwide Children’s Hospital QIS Department, the CAC Mental Health Advocates, and Gail Hornor for their commitment to the implementation of this project and to ensuring that the families receive needed assistance and support. The authors would also like to thank the SEEK Model developers for their assistance in the early implementation phases.
